# Treatment of chronic urticaria with traditional Chinese medicine: A systematic review, meta-analysis, and medication regularity

**DOI:** 10.1097/MD.0000000000042819

**Published:** 2025-06-13

**Authors:** Ruyu Chen, Zuxi Cen, Keyao Zheng, Yu Chen, Tiejun Chen, Chujie Chen

**Affiliations:** a Guangzhou University of Chinese Medicine, Guangzhou, Guangdong Province, China; b Dongguan Qishi Town Community Health Service Center, Dongguan, Guangdong Province, China.

**Keywords:** Chronic urticaria, meta-analysis, therapeutic effect, traditional Chinese medicine

## Abstract

**Background:**

Traditional Chinese medicine (TCM) is often used to treat chronic urticarial (CU), so its efficacy and safety on CU were studied according to systematic review and meta-analysis.

**Methods:**

By searching PubMed, Wanfang, CNKI, and other databases for the literature on the treatment of CU with TCM in the past 10 years (2013–2023), the PICOS principle was used as the criterion for inclusion and exclusion of literature, and the quality of the included literature studies was systematically reviewed according to the Cochrane systematic review method and software RevMan5.3.

**Results:**

A total of 14 articles were included, including 1192 patients. According to the analysis results, the total effective rate of TCM in the treatment of CU was (RR = 1.23, 95% confidence interval (CI) [1.16–1.29], *P* < .00001), the incidence of adverse reactions and recurrence rates were (RR = 0.23, 95% CI [0.13–0.39], *P* < .00001) and (RR = 0.24, 95% CI [0.13–0.43], *P* < .00001), and the symptom score was (MD = −0.38, 95% CI [−0.81–0.05], *P* = .08). The most frequently used Chinese herbs were Windbreak, Licorice, Angelica sinensis, Schizonepeta, Cicada slough, Cassia twig, Astragalus membranaceus, schisandra chinensis, Cortex dictamni and Dark plum.

**Conclusion:**

TCM has great advantages in the treatment of CU, can effectively improve the symptoms, the curative effect is stable and safe, but there are still some deficiencies in the standardization of curative effect evaluation.

## 1. Introduction

Urticaria is a reactive disease characterized by the appearance of wheals, angioedema, or a combination of both symptoms.^[1]^ The wheals present with peripheral erythema and central swelling of variable size, accompanied by intense itching or burning sensation, and are transient. Angioedema appears in the deep dermis and at the boundaries of the subcutaneous or mucous membranes and presents as a pain or burning sensation without pruritus, develops slowly and may last several days.^[2]^ According to the duration of urticaria, it can be classified into acute urticaria and chronic urticaria (CU). CU is characterized by persistent or intermittent urticaria symptoms for more than 6 weeks, and even the course of the disease can be as long as months to years or even decades. Its symptoms and signs are caused by the activation and degranulation of skin mast cells and released mediators,^[3]^ and nearly 1.4% of the population suffer from this disease for life.^[2]^ In urticaria guidelines published in different countries, it is recommended to use a single conventional dose of second-generation nonsedative H1 antihistamine as the first-line treatment for CU.^[4]^ In clinical practice, antihistamines or combined with other drugs are often used as treatment methods. Although the onset of effect is quick, there are certain side effects, and it is easy to develop drug resistance, and the recurrence rate is high after drug withdrawal.

Traditional Chinese medicine (TCM) has a long and profound history in treating CU. The understanding of urticaria in ancient Chinese medicine can be traced back to the Spring and Autumn Period, when it was called “addiction rash.” Its name comes from the bright red or pale wheals that appear on the skin. These wheals appear and disappear, hence the name addiction rash. According to the summary of ancient literature research by medical experts of past dynasties, urticaria is mostly caused by exogenous 6 obscene evil qi, improper diet and internal emotional injuries.^[5]^ Different doctors have formed different treatment rules for CU according to its complex etiology and pathogenesis. For example, using the theory of “subduing blood stasis,” the treatment method of “benefiting yin and nourishing blood, searching collaterals and penetrating blood stasis” is put forward^[6]^; The treatment mainly focuses on clearing away heat and removing dampness and relieving exterior rash, promoting blood circulation and removing blood stasis^[7]^; According to the viewpoint of “treating wind first treats blood, and blood moves wind and destroys itself,” CU is treated from wind and blood theory.^[8]^ Studies have shown that many traditional Chinese medicine preparations can effectively relieve the itching symptoms of urticaria,^[9]^ such as Guizhi Decoction, Yupingfeng Powder, Mahuang Forsythia Chixiaodou Decoction, etc, which have clinical anti-inflammatory, antipruritic and immune regulatory effects.^[10]^ Great progress has been made in the clinical treatment of CU with TCM. Now the efficacy and safety of TCM on CU are systematically evaluated, so as to provide more reliable medical evidence for the clinical treatment of CU with TCM.

## 2. Information and methodology

### 2.1. Sources

#### 2.1.1. Literature inclusion criteria

The subjects were CU patients aged 18 to 65 years who met the diagnostic criteria of Chinese and Western medicine; The intervention measures were that the treatment group was treated with traditional Chinese medicine decoction, while the control group was treated with conventional western medicine, such as loratadine, cetirizine, ebastine and other drugs; The outcome index was the effective rate determined according to the evaluation criteria of therapeutic effect, and the effective rate was obtained by calculating the ratio of the number of cured cases, the number of obvious cases and the number of effective cases in the total number of cases. All the studies were randomized controlled trials.

#### 2.1.2. Exclusion criteria

① Repeated reports; ② Reviews, conference articles, animal experiments, the experience of famous authors, and literature whose full text cannot be obtained.

### 2.2. Methodology

#### 2.2.1. Data retrieval

The computer system searched Wanfang database, VIP journals, CNKI, Cochrane, Pubmed and Embase databases for the last 10 years, from 2013 to 2023; Keywords were CU, urticaria, traditional Chinese medicine, traditional Chinese medicine, random. Preliminary acquisition of 550 Chinese literature and 42 English literature. Taking PubMed as an example, the retrieval formula is as follows. See Table 1 for details.

**Table 1:** Strategies in PubMed.

ID	Query
**#1**	“chronic urticaria”[MeSH Terms]
**#2**	“chronic urticarias”[Title/Abstract] OR “urticaria chronic”[Title/Abstract] OR “chronic spontaneous urticaria”[Title/Abstract] OR (((“Chronic”[All Fields] OR “chronical”[All Fields] OR “chronically”[All Fields] OR “chronicities”[All Fields] OR “chronicity”[All Fields] OR “chronicization”[All Fields] OR “chronics”[All Fields]) AND (“Spontaneous”[All Fields] OR “spontaneously”[All Fields])) AND “Urticarias”[Title/Abstract]) OR “spontaneous urticaria chronic”[Title/Abstract] OR “urticaria chronic spontaneous”[Title/Abstract] OR “idiopathic chronic urticaria”[Title/Abstract] OR “chronic urticaria idiopathic”[Title/Abstract] OR ((“Idiopathic”[All Fields] OR “idiopathically”[All Fields] OR “idiopathics”[All Fields]) AND “chronic urticarias”[Title/Abstract]) OR ((“Urticaria”[MeSH Terms] OR “Urticaria”[All Fields] OR “Urticarias”[All Fields]) AND “idiopathic chronic”[Title/Abstract]) OR “chronic idiopathic urticaria”[Title/Abstract] OR ((“Chronic”[All Fields] OR “chronical”[All Fields] OR “chronically”[All Fields] OR “chronicities”[All Fields] OR “chronicity”[All Fields] OR “chronicization”[All Fields] OR “chronics”[All Fields]) AND “idiopathic urticarias”[Title/Abstract]) OR “idiopathic urticaria chronic”[Title/Abstract] OR “urticaria chronic idiopathic”[Title/Abstract] OR “autoimmune urticaria”[Title/Abstract] OR ((“Autoimmune”[All Fields] OR “autoimmunity”[MeSH Terms] OR “autoimmunity”[All Fields] OR “autoimmunities”[All Fields] OR “autoimmunization”[All Fields] OR “autoimmunizing”[All Fields]) AND “Urticarias”[Title/Abstract]) OR “urticaria autoimmune”[Title/Abstract] OR “chronic autoimmune urticaria”[Title/Abstract] OR “autoimmune urticaria chronic”[Title/Abstract] OR (((“Chronic”[All Fields] OR “chronical”[All Fields] OR “chronically”[All Fields] OR “chronicities”[All Fields] OR “chronicity”[All Fields] OR “chronicization”[All Fields] OR “chronics”[All Fields]) AND (“Autoimmune”[All Fields] OR “autoimmunity”[MeSH Terms] OR “autoimmunity”[All Fields] OR “autoimmunities”[All Fields] OR “autoimmunization”[All Fields] OR “autoimmunizing”[All Fields])) AND “Urticarias”[Title/Abstract]) OR “urticaria chronic autoimmune”[Title/Abstract]
**#3**	#1 OR #2
**#4**	“medicine, chinese traditional”[MeSH Terms]
**#5**	“zhong yi xue”[Title/Abstract] OR ((chung i[Author] OR chung i[Investigator]) AND “Hsueh”[Title/Abstract]) OR (“Hsueh”[All Fields] AND “chung i”[Title/Abstract]) OR “traditional medicine chinese”[Title/Abstract] OR “chinese traditional medicine”[Title/Abstract] OR “traditional chinese medicine”[Title/Abstract] OR “chinese medicine traditional”[Title/Abstract] OR “traditional tongue diagnosis”[Title/Abstract] OR (((“Tongue”[MeSH Terms] OR “Tongue”[All Fields] OR “tongues”[All Fields] OR “tongue s”[All Fields]) AND (“diagnosable”[All Fields] OR “diagnosi”[All Fields] OR “Diagnosis”[MeSH Terms] OR “Diagnosis”[All Fields] OR “diagnose”[All Fields] OR “diagnosed”[All Fields] OR “Diagnoses”[All Fields] OR “diagnosing”[All Fields] OR “Diagnosis”[MeSH Subheading])) AND “Traditional”[Title/Abstract]) OR (((“Tongue”[MeSH Terms] OR “Tongue”[All Fields] OR “tongues”[All Fields] OR “tongue s”[All Fields]) AND (“diagnosable”[All Fields] OR “diagnosi”[All Fields] OR “Diagnosis”[MeSH Terms] OR “Diagnosis”[All Fields] OR “diagnose”[All Fields] OR “diagnosed”[All Fields] OR “Diagnoses”[All Fields] OR “diagnosing”[All Fields] OR “Diagnosis”[MeSH Subheading])) AND “Traditional”[Title/Abstract]) OR ((“tradition”[All Fields] OR “tradition s”[All Fields] OR “Traditional”[All Fields] OR “traditionals”[All Fields] OR “traditions”[All Fields]) AND “tongue diagnoses”[Title/Abstract]) OR ((“tradition”[All Fields] OR “tradition s”[All Fields] OR “Traditional”[All Fields] OR “traditionals”[All Fields] OR “traditions”[All Fields]) AND “tongue assessment”[Title/Abstract]) OR ((“Tongue”[MeSH Terms] OR “Tongue”[All Fields] OR “tongues”[All Fields] OR “tongue s”[All Fields]) AND “assessment traditional”[Title/Abstract]) OR ((“tradition”[All Fields] OR “tradition s”[All Fields] OR “Traditional”[All Fields] OR “traditionals”[All Fields] OR “traditions”[All Fields]) AND “tongue assessments”[Title/Abstract])
**#6**	#4 OR #5
**#7**	#3 AND #6
**#8**	#7 AND (y_10[Filter])

#### 2.2.2. Literature screening and data extraction

Use a computer to integrate literature, with 2 independent data analysts conducting the integration. Firstly, use NoteExpress software to remove duplicate literature. Secondly, read the titles and abstracts of the literature to remove non compliant literature according to the exclusion criteria, such as reviews, animal experiments, expert experiences, etc. Then, by reading the entire text, remove non compliant literature such as intervention measures and age. If there are any objections to the literature, they will be discussed by 2 people or contacted by a third party to determine the final inclusion of the literature. And extract and include literature data information, including author names, intervention measures, outcome indicators, and other necessary data.

In addition, it is necessary to extract information on the use of TCM from literature. For literature on adjusting medication according to symptoms, 29 traditional Chinese medicine prescriptions should be extracted based on the principle of “one group of main drugs + one group of dispensing drugs = one Chinese medicine prescription.” Refer to “Chinese Materia Medica”^[11]^ and the first part of the 2020 edition of “Pharmacopoeia of the People’s Republic of China”^[12]^ to standardize the names of Chinese medicines, and input the standardized information of Chinese medicines into Excel 2021 to establish a database.

#### 2.2.3. Quality evaluation and statistical analysis

Two researchers used the Cochrane Risk Bias Assessment tool to evaluate the quality of the included randomized controlled studies, and assessed the risk by evaluating the method of randomization, whether blinding method was used, and whether selective reporting was performed, etc. Based on this, the literature is divided into 3 grades: “high risk of bias,” “low risk of bias” and “unclear risk of bias.”

The included literature was analyzed using Revman5.3 software, with relative risk with 95% confidence interval (CI) for binary variables and mean standard deviation (MD) with 95% CI for continuous variables. Cochran *Q* and I statistic tests were used to assess statistical heterogeneity. I^2^ statistics preliminarily divide 25% to 50%, 51% to 75% and 76% to 100% into low, medium and high heterogeneity respectively. When I^2^ < 50%, it is considered that the heterogeneity among the studies is small, and the fixed effects model is used for analysis; I^2^ > 50% indicates significant heterogeneity among the studies, and random effects model is used for heterogeneity treatment and then meta-analysis is performed. Funnel plots were used to detect any publication bias.

#### 2.2.4. Statistical methods

Collect the frequency information of TCM usage in Excel 2021, use IBM SPSS MODELER 18.0 to analyze the association rules of all traditional Chinese medicines in the prescription, and use IBM SPSS Statistics statistical software to conduct systematic clustering analysis of high-frequency traditional Chinese medicines. When analyzing all traditional Chinese medicines using association rules, the Apriori algorithm is used. The filtering criteria are set to a maximum of 2 leading terms, a minimum rule confidence of 80%, and a minimum condition support of 10%. The charts are drawn to analyze the correlation between the compatibility of traditional Chinese medicines.

## 3. Results

### 3.1. Literature retrieval

**Figure 1.**
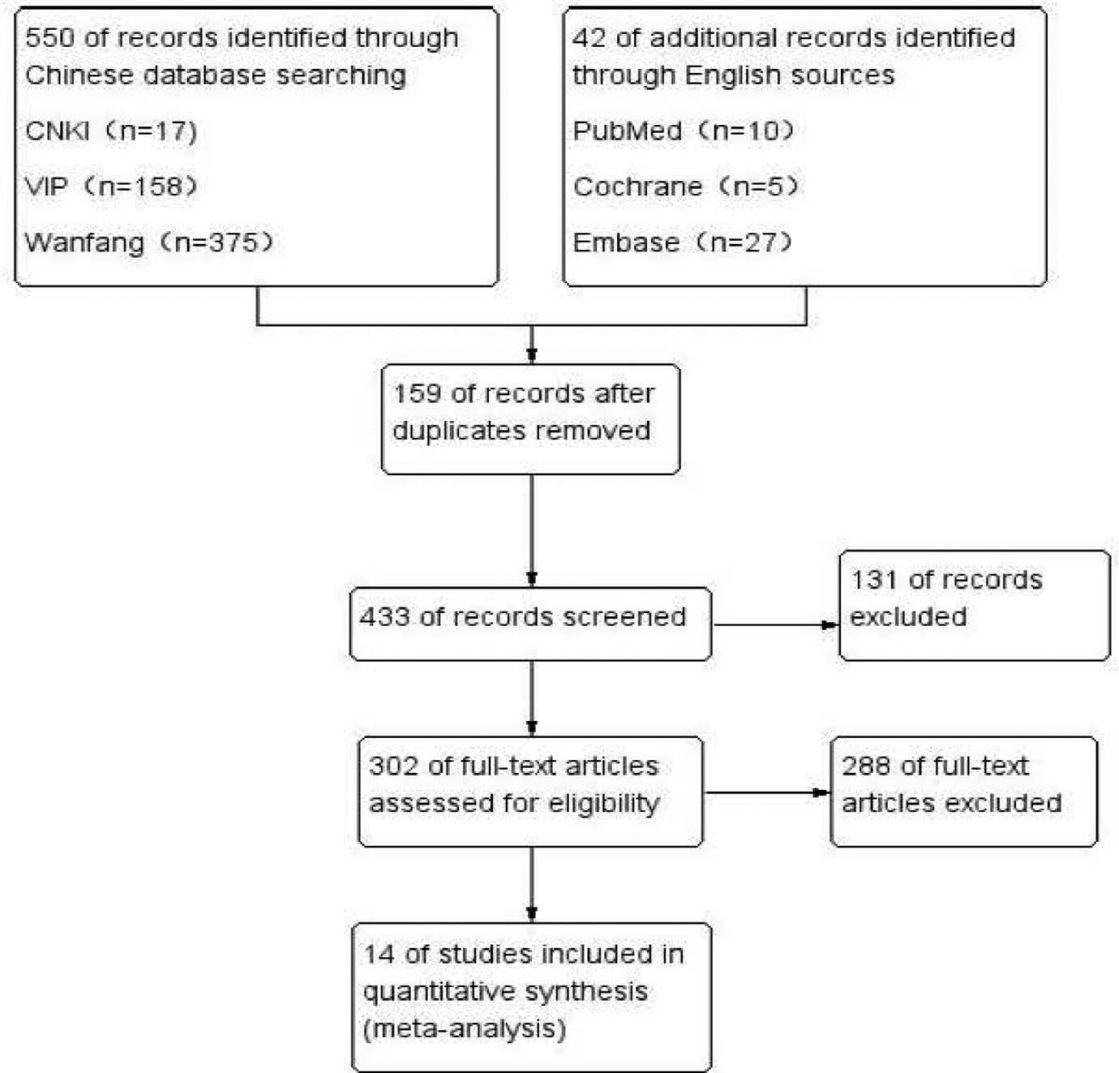
Literature screening process. CNKI = China national knowledge infrastructure.

According to the search strategy, a preliminary retrieval of the database was conducted to obtain 550 Chinese literature and 42 English literature. Using NoteExpress software, 159 duplicate articles were removed, and 131 articles that did not meet the inclusion criteria were excluded by reading the titles and abstracts of the articles. Then, by reading the full text, 288 articles that did not meet the inclusion criteria were found. Finally, 14 articles were included. Refer to Figure 1 for details.

### 3.2. Basic information of literature

This study included a total of 14 articles, all of which were from China. A total of 1192 patients were involved, including 604 in the TCM group and 588 in the control group. All literature reports detailed intervention measures, including usage, dosage, and course of treatment, with the control group mainly using antihistamines according to clinical guidelines. In terms of disease progression, there are 4 articles^[13–16]^ in years and 6 articles^[17–22]^ in months, and the rest of the articles are not mentioned. In terms of treatment course, the treatment course of 4 articles^[14,19,20,22]^ was 8 weeks; The treatment course for 4 articles^[13,17,18,23]^ was 4 weeks; The treatment course for 2 articles^[15,21]^ was 1 month; The treatment courses for the other 4 articles^[16,24–26]^ were 28 days, 20 days, 15 days, and 3 weeks, respectively. All literature includes clinical efficacy rates, with 7 literature^[13,14,16,17,21,22,25]^ reporting adverse reactions, 3 literature^[16,17,20]^ reporting recurrence, and 4 literature^[15,18,20,22]^ using symptom scores. See Table 2 for details.

**Table 2:** Basic characteristics of included literature.

Author	Year	Gender		Age ($\bar{x}\pm \;s$, yr)		Course of illness ($\bar{x}\pm \;s$)		Treatment course	Intervention measures	
		(Male/female)								
		Treatment group	Control group	Treatment group	Control group	Treatment group	Control group		Treatment group	Control group
Chen^[18]^	2018	24/18	27/15	32.76 ± 5.98	32.49 ± 6.12	2.45 ± 0.43	2.61 ± 0.47 mo	4 wk	Yang-He decoction modified	Cetirizine
Sun^[20]^	2021	12/33	15/31	42.0 ± 6.4	43.0 ± 5.4	18.0 ± 5.5	18.2 ± 5.6 mo	8 wk	Yangxue Qufeng decoction	Olatadine hydrochlorid e tablets
Lu^[19]^	2020	23/21	24/21	40.01 ± 7.34	39.25 ± 7.12	31.13 ± 5.86	30.24 ± 6.12 mo	8 wk	Astragalus and Guizhi Wuwu decoction with Allergic decoction	Ebastine tablets
Zhu^[22]^	2021	20/28	21/27	36.42 ± 10.83	35.06 ± 11.65	21.43 ± 12.41	20.27 ± 13.68 mo	8 wk	Yinlian Qufeng Decoction	Desloratadine dispersible tablets
Zhang^[21]^	2021	20/28	21/27	36.42 ± 10.83	44.39 ± 2.15	9.25 ± 1.25	9.51 ± 1.62 mo	1 mo	Guizhi– decoction modified	levocetirizine hydrochloride
Dong^[13]^	2020	15/15	16/14	27.46 ± 2.56	27.89 ± 2.67	4.68 ± 2.32	4.67 ± 2.78 yr	4 wk	Guizhi decoction and Yupingfeng Powder modified	Terfenadine tablets
Fei ^16]^	2019	25/23	26/22	32.8 ± 11.5	31.5 ± 10.2	5.1 ± 2.4	4.7 ± 2.6 yr	15 d	Four ingredient decoction with allergic decoction modified	Ebastinpine
Chen^[17]^	2016	14/16	15/15	35.3 ± 1.2	35.2 ± 1.2	24.5 ± 1.2	24.2 ± 1.1 mo	4 wk	Modified Shen Ling Bai Zhu powder	Loratadine tablet
Wang^[14]^	2016	36/34	34/36	32.5 ± 15.6	31.5 ± 16.3	2.6 ± 2.1	2.4 ± 2.2 yr	8 wk	Modified Dehumidification Weiling decoction	Desloratadine tablets + Chlorpheniramine Maleate Tablets
Wang^[25]^	2014	22/20	18/24	33.1	32.5	Unknown	Unknown	28 d	Guizhi– Decoction modified	Chlorpheniramine Maleate Tablets + Cetirizine
Xu^[15]^	2013	29/31	22/27	38.12 ± 9.28	37.55 ± 10.23	0.92 ± 0.81	0.96 ± 0.87 yr	1 mo	Urticaria decoction	Loratadine
Li^[24]^	2016	Unknown	Unknown	Unknown	Unknown	Unknown	Unknown	20 d	Chinese medicine	Loratadine
Xiao^[26]^	2014	Unknown	Unknown	Unknown	Unknown	Unknown	Unknown	3 wk	Yupingfeng powder modified	Ebastine + doxepin hydrochloride
Zhao^[23]^	2013	Unknown	Unknown	Unknown	Unknown	Unknown	Unknown	4 wk	Guifeng decoction	Cetirizine hydrochloride tablet

### 3.3. Quality evaluation

**Figure 2.**
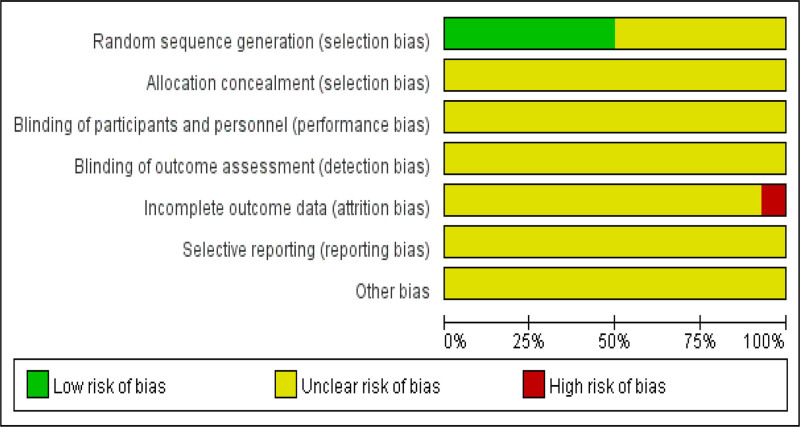
Inclusion of literature quality evaluation.

The risk assessment of literature quality is shown in Figure 2.

① Random sequence generation: 7 studies^[14–18,20,21]^ were evaluated as low risk of bias using the random number table method, while the rest of the studies only described random assignment, and the method was unknown and evaluated as unclear risk of bias.
② Assignment concealment: All studies did not mention the assignment concealment method, which was evaluated as unclear risk of bias.
③ Blind method: None of the studies mentioned blind method, and the risk of bias was assessed as unclear.
④ Data integrity: 13 studies^[13–19,21–26]^ did not specifically report the relevant situation, and the risk of bias was evaluated as unclear, and 1 study^[20]^ was evaluated as high risk of bias with cases lost to follow-up.
⑤ Selective reporting: All studies were evaluated as unclear risk of bias.
⑥ Other bias: All studies were evaluated as unclear risk of bias.

### 3.4. Meta-analysis results

#### 3.4.1. Total effective rate

**Figure 3.**
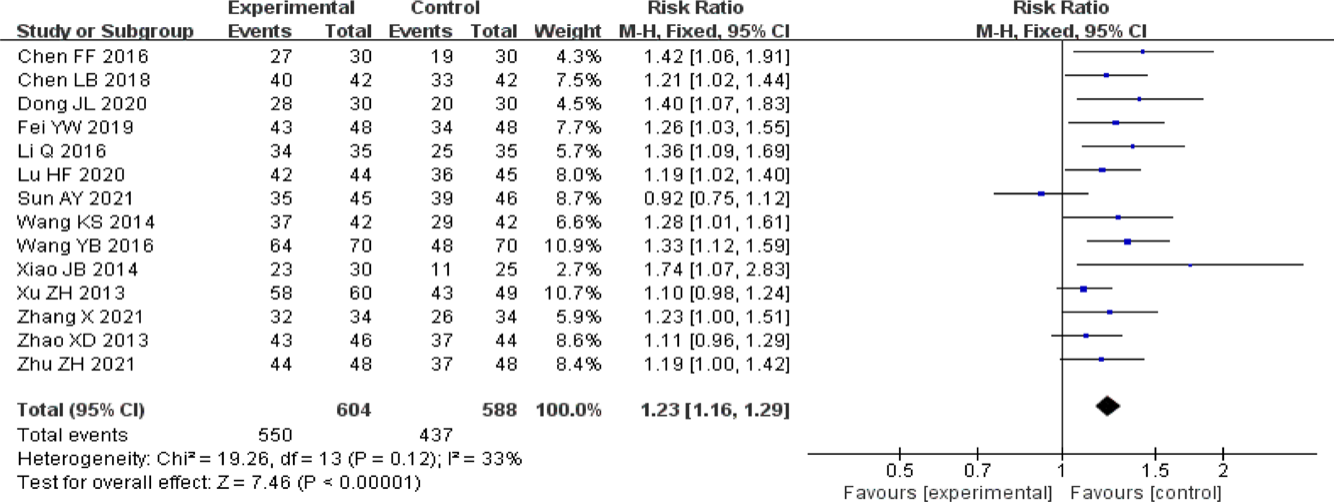
Forest plot of clinical total effective rate. CI = confidence interval.

**Figure 4.**
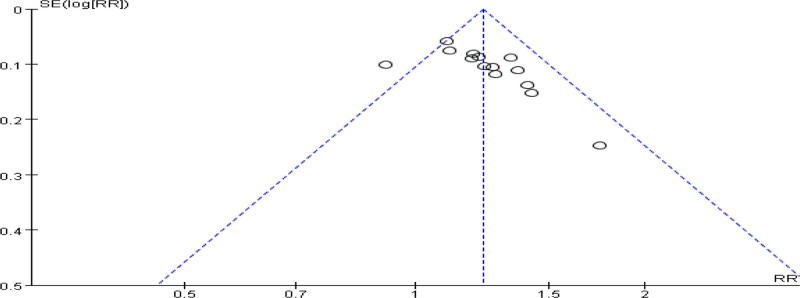
Publication bias funnel plot. RR = relative risk.

All studies reported effective rates. The heterogeneity evaluation through I^2^ statistical test found that there was low heterogeneity in the study (I^2^ = 33%). The results obtained by fixed-effect model analysis: the clinical effectiveness of TCM in the treatment of CU was higher than that of western medicine, and the difference was statistically significant (RR = 1.23, 95% CI [1.16–1.29], *P* < .00001). See Figure 3 for details. A funnel plot analysis of the publication bias of the total response rate of the included literature is shown in Figure 4. The funnel plot shows poor left-right symmetry and falls somewhat outside the CI line, suggesting some publication bias.

#### 3.4.2. Adverse reaction incidence rate

**Figure 5.**
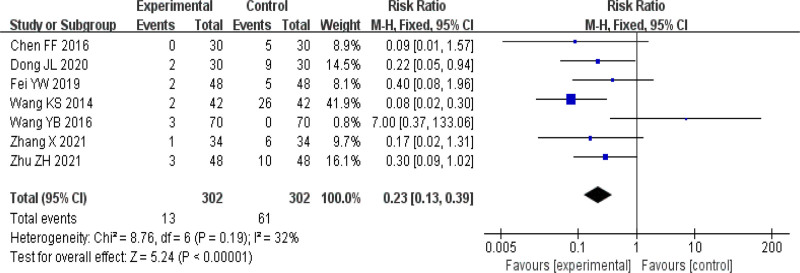
Forest plot of adverse reactions. CI = confidence interval.

A total of 7 articles^[13,14,16,17,21,22,25]^ reported the occurrence of adverse reactions. In these 7 articles, there were a total of 13 adverse reactions in the TCM treatment group, mainly manifested as gastrointestinal discomfort, while the control group had a total of 61 adverse reactions, mainly manifested as dry mouth, drowsiness, fatigue, and other symptoms. After analyzing the adverse reaction data, it was found (I^2^ = 32%) that the heterogeneity of the study was small. Using a fixed effects model analysis, the results showed that the incidence of adverse reactions in the treatment of CU with TCM was lower, and the difference was statistically significant (RR = 0.23, 95% CI [0.13, 0.39], *P* < .0001). See Figure 5 for details.

#### 3.4.3. Recurrence rate

**Figure 6.**
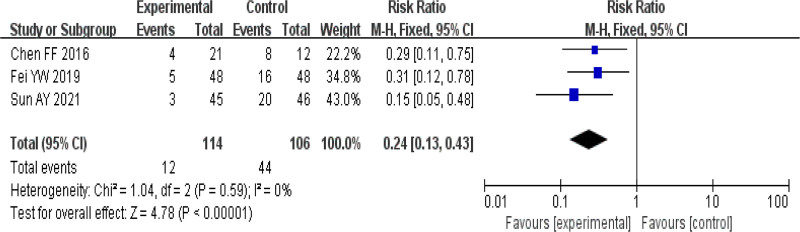
Forest plot of recurrence rate. CI = confidence interval.

Only 3 articles^[16,17,20]^ reported recurrence. Two of them^[16,20]^ followed up all patients and calculated the recurrence rate, while the other^[17]^ calculated the recurrence rate by follow-up of those who were cured. The rest of the studies were not mentioned. The recurrence rate was analyzed using a fixed effects model with I^2^ = 0%, and it was found that the TCM group had a positive effect in reducing the recurrence of CU, and the recurrence rate was lower than that of the control group, and the results were statistically significant (RR = 0.24, 95% CI [0.13, 0.43], *P* < .00001). See Figure 6.

#### 3.4.4. Symptom score

**Figure 7.**
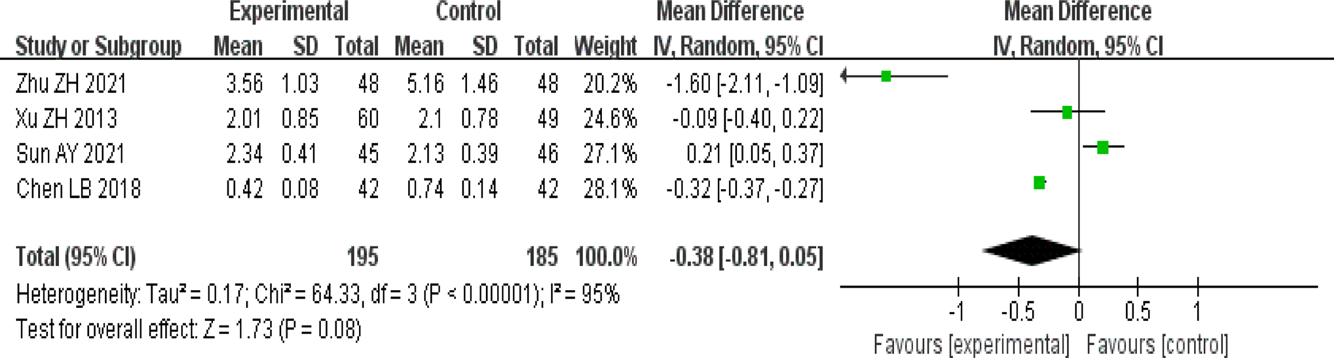
Forest plot of symptom score. CI = confidence interval, IV = inverse-variance weighting, SD = standard deviation.

1 article^[21]^ only describes the condition of wheal mass; 3 articles^[17,23,25]^ described the 4-level scoring rule, but did not record the data; 2 articles^[16,22]^ recorded TCM symptom scores; An analysis was conducted on 4 articles^[15,18,20,22]^ that recorded symptom scores before and after treatment, and the results showed that the heterogeneity among the studies was significant (I^2^ = 95%). Using random effects model analysis, the difference was not statistically significant (MD = −0.38, 95% CI [−0.81 to 0.05], *P* = .08). See Figure 7. In order to evaluate the stability of the results of this study, the pooled analysis was performed after excluding individual studies one by one in Revman5.3 software, and the results showed that the pooled results changed from statistically significant to statistically significant after excluding Sun^[20]^ study (MD = −0.61, 95% CI [−1.18 to −0.04], *P* = .04).

### 3.5. Situation of Chinese medicine use

#### 3.5.1. High-frequency herbs in Chinese medicine prescriptions

The 29 Chinese medicine prescriptions extracted from 14 articles included 95 Chinese medicines, with a total use frequency of 410 times. If the condition for high-frequency TCM is set to be used more than 8 times, it includes 16 traditional Chinese medicines, among which the top 10 are Windbreak, Licorice, Angelica sinensis, Schizonepeta, Cicada slough, Cassia twig, Astragalus membranaceus, schisandra chinensis, Cortex dictamni and Dark plum; The usage frequencies are 27 times, 23 times, 21 times, 19 times, 14 times, 13 times, 13 times, 13 times, 12 times, and 12 times respectively, as shown in Table 3.

**Table 3:** High frequency drugs in Traditional Chinese Medicine prescriptions (use times > 8 times).

Serial number	Chinese medicine	Frequency (times)	Frequency* (%)
**1**	Windbreak	27	93.1
**2**	Licorice	23	79.31
**3**	Angelica sinensis	21	72.41
**4**	Schizonepeta	19	65.52
**5**	Cicada slough	14	48.28
**6**	Cassia twig	13	44.83
**7**	Astragalus membranaceus	13	44.83
**8**	Schisandra chinensis	13	44.83
**9**	Cortex dictamni	12	41.38
**10**	Dark plum	12	41.38
**11**	White peony	10	34.48
**12**	Ligusticum wallichii	10	34.48
**13**	Rehmannia glutinosa	10	34.48
**14**	Tribulus terrestris	10	34.48
**15**	Atractylodes macrocephala	9	31.03
**16**	Kochia scoparia	9	31.03

* Frequency = Frequency of application of Chinese medicine/total number of Chinese medicine prescriptions (29).

#### 3.5.2. Analysis of herb association rules

**Figure 8.**
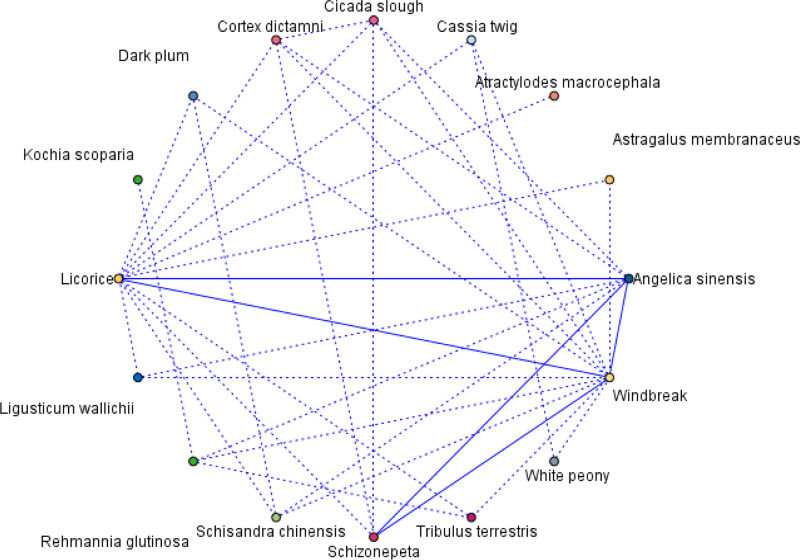
Network diagram of drug association rules.

According to the association rule chart drawn by IBM SPSS MODELER 18.0 software, there are 5 pairs of moderate links displayed in the network chart, namely Windbreak – Licorice 22 times, Angelica sinensis – Windbreak 21 times, Windbreak – Schizonepeta 18 times, Angelica sinensis – Schizonepeta 17 times, and Angelica sinensis – Licorice 16 times, as shown in Figure 8. Select association rules based on support percentage > 45%, as shown in Table 4. Among them, the combination of Windbreak – Licorice, Licorice – Windbreak, Angelica sinensis – Schizonepeta, Angelica sinensis – Windbreak, Angelica sinensis – Windbreak – Schizonepeta ranks among the top 5.

**Table 4:** Drug association rules (support > 45%).

Subsequent item	Preceding item	Examples	Support percentage (%)	Confidence percentage (%)
Licorice	Windbreak	27	93.1	81.48
Windbreak	Licorice	23	79.31	95.65
Schizonepeta	Angelica sinensis	21	72.41	80.95
Windbreak	Angelica sinensis	21	72.41	100
Schizonepeta	Angelica sinensis and Windbreak	21	72.41	80.95
Angelica sinensis	Schizonepeta	19	65.52	89.47
Windbreak	Schizonepeta	19	65.52	94.74
Angelica sinensis	Schizonepeta and Windbreak	18	62.07	94.44
Windbreak	Schizonepeta and Angelica sinensis	17	58.62	100
Windbreak	Angelica sinensis and Licorice	16	55.17	100
Schizonepeta	Cicada slough	14	48.28	92.86
Licorice	Cicada slough	14	48.28	100
Windbreak	Cicada slough	14	48.28	92.86
Schizonepeta	Cicada slough and Licorice	14	48.28	92.86
Cicada slough	Schizonepeta and Licorice	14	48.28	92.86
Windbreak	Cicada slough and Licorice	14	48.28	92.86
Angelica sinensis	Schizonepeta and Licorice	14	48.28	85.71
Windbreak	Schizonepeta and Licorice	14	48.28	92.86

#### 3.5.3. High-frequency herb cluster analysis

**Figure 9.**
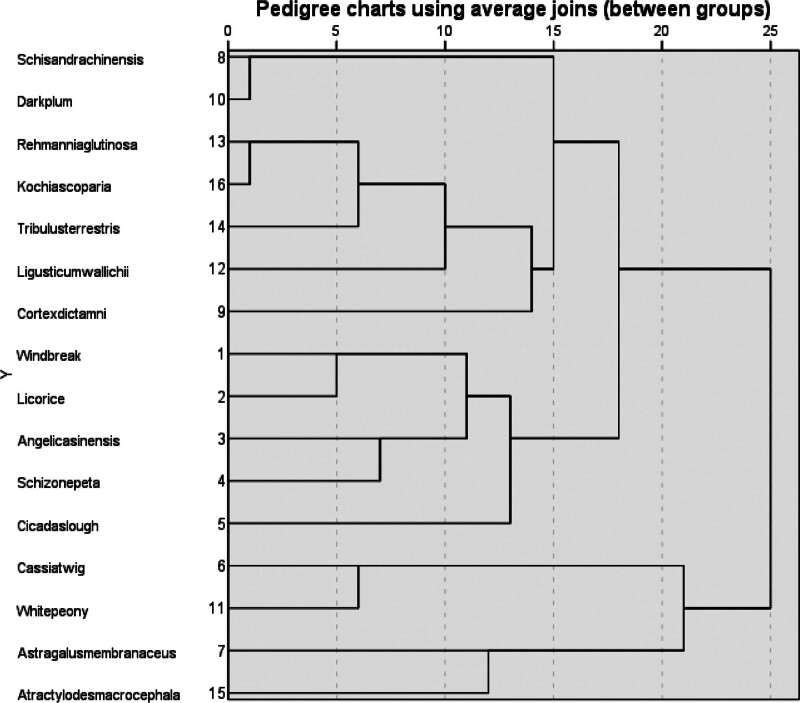
Cluster analysis of high-frequency drugs in traditional Chinese medicine prescriptions.

Import all traditional Chinese medicines into IBM SPSS Statistics software for cluster analysis, using inter group connections. The results are shown in Figure 9 of the pedigree chart. If the pedigree chart is divided according to a scale of 20, 3 categories can be obtained, namely: Class: Schisandra chinensis, Dark Plum, Rehmannia glutinosa, Kochia scoparia, Tribulus terrestris, Ligusticum wallichii, Cortex dictamni, Windbreak, Licorice, Angelica sinensis, Schizonepeta, Cicada slough; Class: Cassia twig, White peony; Class: Astragalus membranaceus, Atractylodes macrocephala.

## 4. Discussion

The analysis of this study concluded that compared with antihistamines, TCM has a higher clinical success rate, lower recurrence rate and lower occurrence of adverse events. The total symptom score rating was comparable between the 2 groups.

CU is a common skin disease, which is prone to allergic constitution and people with insufficient endowment. Meta-analysis shows that TCM has higher clinical efficacy on this disease. Through dialectical analysis of TCM during the treatment process, dialectical medication for different people’s constitution, etiology and disease can better promote patients’ physical rehabilitation and improve their quality of life. Sun Mingxin^[27]^ believe that the imbalance of the opening and closing of sweat pores in the skin caused by various reasons is an important cause of this disease, and the functions of skin, lung, spleen, etc are closely related to the normal opening and closing of sweat pores. Insufficient Wei Qi cannot control the normal opening and closing of sweat pores, and when it is not closed, it leads to disharmony between the Ying and Wei systems, and external evil easily enters and causes disease.^[28]^ Some scholars^[10]^ have found that the pharmacological mechanism of TCM in the treatment of CU is mainly to inhibit allergic reaction mechanism and degranulation of immune cells. For example, Yupingfeng Powder has been proved to be effective for type I allergic diseases, and can inhibit the production of IgE and the release of histamine by mast cells.^[29]^ This article analyzed the use of formulas in 14 included literature and found that Guizhi Decoction, Yupingfeng Powder and Yangxue Qufeng Decoction are the main formulas with the effects of tonifying qi dispelling wind and relieving itching. In terms of the frequency of use of TCM, Windbreak, Licorice, Angelica sinensis, Schizonepeta, Cicada slough, Cassia twig, Astragalus membranaceus, schisandra chinensis, Cortex dictamni and Dark plum are the main herbs. By using Windbreak, Schizonepeta, and Cassia twig to dispel external pathogens, Cortex dictamni can relieve dampness and itching, White peony combined with Cassia twig can regulate the body’s health, and Angelica sinensis can promote blood circulation to dispel wind, thus playing a therapeutic role. Based on the association rules and clustering analysis results, it can be concluded that the treatment of CU mainly focuses on dispelling wind and relieving itching, supplemented by harmonizing the body’s health and nourishing the vital energy.

For the treatment plan of CU, antihistamines are the first choice for the first-line treatment plan. If the symptoms continue to not improve, omalizumab, cyclosporine, montelukast and other treatments can be used as appropriate, but adverse reactions such as liver and kidney damage are prone to occur.^[30]^ After meta-analysis, it was found that the experimental group was better than the control group in reducing the recurrence rate and adverse reaction rate. Adverse reactions are not easy to occur in the treatment of CU with TCM, and symptoms such as dry mouth and mild nausea can be relieved by themselves without medication.

In terms of improving symptoms, when the symptom scores were analyzed in the TCM group and the antihistamine group, it was found that the combined results changed from nonstatistically significant to statistically significant after excluding Sun^[20]^ study. Through analysis, it is found that this result may be due to the inconsistency of symptom score evaluation criteria used in the literature using symptom score. In addition, the efficacy evaluation criteria of CU^[31]^ currently include: 7-day urticaria activity score (UAS7), urticaria control score (UCT), dermatology quality of life index (DLQI) and CU quality of life questionnaire (CU-Q2oL) and other standard scales, but only 1 eligible literature^[22]^ used the DLQI scale and one^[20]^ used the UAS scale for evaluation; Other literatures use the changes of clinical symptoms before and after treatment under different standards to observe and evaluate the efficacy level, which affects the rigor of its efficacy evaluation to a certain extent. There is no uniform reporting standard for the outcome indicators of similar clinical trials, which makes it difficult to summarize clinical evidence.^[32]^ In order to make the clinical research results comparable and practical, especially in the clinical research of TCM, it is urgent to establish a core index set to solve this problem.^[33]^ At present, there is no clear evidence to show whether the differences caused by inconsistent evaluation criteria are significant, and more high-quality studies are needed to verify the results.

Through the systematic analysis of literature, TCM has great advantages in the treatment of CU, which can effectively improve symptoms, and has stable and safe curative effect, but there are certain limitations. The implementation of blinding method was not specified in the study design of the literature included in this study, and some literatures did not specify the way of generating randomization, which may affect the rigor of the study results. The included literature lacks long-term follow-up data, and some literatures are not evaluated by recognized evaluation criteria.

## 5. Summary

This meta-analysis shows that compared with antihistamines, TCM has better efficacy and higher safety in the treatment of CU. However, due to the limitations of this study, it is necessary to carry out more large sample sizes and high-quality studies in different regions in future clinical studies, promote the use of recognized evaluation standards for CU to verify conclusions, and increase the reliability of evaluation of the efficacy of TCM in the treatment of CU.

## Author contributions

**Conceptualization:** Ruyu Chen.

**Data curation:** Ruyu Chen, Zuxi Cen, Yu Chen.

**Formal analysis:** Keyao Zheng, Tiejun Chen, Chujie Chen.

**Funding acquisition:** Chujie Chen.

**Software:** Ruyu Chen, Keyao Zheng.

**Supervision:** Tiejun Chen, Chujie Chen.

**Validation:** Zuxi Cen, Yu Chen.

**Visualization:** Ruyu Chen.

**Writing – original draft:** Ruyu Chen, Zuxi Cen.

**Writing – review & editing:** Chujie Chen.
